# Constrained groupwise additive index models

**DOI:** 10.1093/biostatistics/kxac023

**Published:** 2022-07-06

**Authors:** Pierre Masselot, Fateh Chebana, Céline Campagna, Éric Lavigne, Taha B M J Ouarda, Pierre Gosselin

**Affiliations:** Department of Public Health, Environment and Society, London School of Hygiene & Tropical Medicine, 15-17 Tavistock Place, WC1H 9SH, London, UK; Centre Eau-Terre-Environnement, Institut National de la Recherche Scientifique, 490, rue de la Couronne, Québec (Québec), G1K 9A9, Canada; Centre Eau-Terre-Environnement, Institut National de la Recherche Scientifique, 490, rue de la Couronne, Québec (Québec), G1K 9A9, Canada and Institut National de Santé Publique du Québec, 945, avenue Wolfe Québec (Québec) G1V 5B3 Canada; School of Epidemiology and Public Health, University of Ottawa, 600 Peter Morand Crescent, Room 101, Ottawa, Ontario K1G 5Z3, Canada and Air Health Science Division, Health Canada, 269 Laurier Avenue West, Mail Stop 4903B, Ottawa, Ontario K1A0K9 Canada; Centre Eau-Terre-Environnement, Institut National de la Recherche Scientifique, 490, rue de la Couronne, Québec (Québec), G1K 9A9, Canada; Institut National de la Recherche Scientifique, Centre Eau-Terre-Environnement, Québec, Canada, Institut National de Santé Publique du Québec, Québec, Canada, and Ouranos, Montréal, 550 Sherbrooke Ouest, Tour Ouest, 19eme Étage, Montréal (Québec), H3A 1B9, Canada

**Keywords:** Additive index models, Dimension reduction, Index, Linear constraints, Quadratic programming

## Abstract

In environmental epidemiology, there is wide interest in creating and using comprehensive indices that can summarize information from different environmental exposures while retaining strong predictive power on a target health outcome. In this context, the present article proposes a model called the constrained groupwise additive index model (CGAIM) to create easy-to-interpret indices predictive of a response variable, from a potentially large list of variables. The CGAIM considers groups of predictors that naturally belong together to yield meaningful indices. It also allows the addition of linear constraints on both the index weights and the form of their relationship with the response variable to represent prior assumptions or operational requirements. We propose an efficient algorithm to estimate the CGAIM, along with index selection and inference procedures. A simulation study shows that the proposed algorithm has good estimation performances, with low bias and variance and is applicable in complex situations with many correlated predictors. It also demonstrates important sensitivity and specificity in index selection, but non-negligible coverage error on constructed confidence intervals. The CGAIM is then illustrated in the construction of heat indices in a health warning system context. We believe the CGAIM could become useful in a wide variety of situations, such as warning systems establishment, and multipollutant or exposome studies.

## 1. Introduction

In environmental epidemiology, there is an increasing recognition of the complexity of the mixture of exposure to human health. The number of exposures, be it pollutants, weather variables, or built-environment characteristics can be numerous and interact in a complex fashion to impact human health. The ever-increasing amount of data available allows for complex statistical and machine learning models to be considered. For instance, recent advances such as Bayesian kernel machine regression (Bobb *and others*, 2015), random forests (Breiman, 2001), or more generally many nonparametric algorithms can achieve impressive predictive power. However, the mentioned models are often referred to as “black boxes” (a recent example is Schmidt, 2020) and are challenging to interpret in practice (e.g., Davalos *and others*, 2017).

Interpretability of model outputs may be a key component of many real-world applications, especially when they involve decision-making or risk assessment (Rudin, 2019). Public health scientists or decision-makers need clear and easy-to-interpret insights about how the different exposures may impact the given health outcome. Examples include weather-related factors (Chebana *and others*, 2013; Pappenberger *and others*, 2015) or air quality indices (Lee *and others*, 2011; Masselot *and others*, 2019). The pool of methods used to create indices is currently limited, as many indices are constructed based on previously estimated univariate risks or created based on a literature review (e.g., Monforte and Ragusa, 2018). Another type of study seeking to summarize a large amount of information, exposome-wide association studies, usually focus on linear methods selecting a few number of exposure, thus partly discarding the complexity of the exposure mixture (e.g., Nieuwenhuijsen *and others*, 2019). Other studies consider index-based methods through the popular weighted quantile sum regression that still relate the created index linearly to the response variable (Keil *and others*, 2020). Therefore, there is a need for methods able to account for complex mixtures of many variables and provide interpretable indices.

Starting from a pool of exposures $\boldsymbol{X} \in \mathbb{R}^d$, indices are defined as a small number $p < d$ of custom predictors $Z$ that are linear combinations of the original predictors, i.e., of the form $Z = \boldsymbol\alpha^T\boldsymbol{X}$. In this sense, deriving indices $Z$ can be seen as a dimension reduction problem. The most famous example of a dimension reduction method is principal component analysis (Jolliffe, 2002). However, in the present work, we are especially interested in a regression context, i.e., in deriving indices related to a response of interest. Methods that are suited for this objective include the single-index model (SIM, e.g., Härdle *and others*, 1993) in which one index is constructed through the model $Y = g(\boldsymbol\alpha^T\boldsymbol{X}) + \varepsilon$ or the projection pursuit regression (PPR, Friedman and Stuetzle, 1981), also known as the additive index model, which extends the SIM in the following fashion: $Y = \sum_j g_j (\boldsymbol\alpha_j^T \boldsymbol{X}) +\varepsilon$. In both models, $g$ and $g_j$ are nonlinear functions representing the relationship between the response $Y$ and the constructed index $Z_j = \boldsymbol\alpha_j^T \boldsymbol{X}$.

Although the SIM and PPR models are often used as nonparametric regression models (Wang and Ni, 2008; Yuan *and others*, 2016; Durocher *and others*, 2016; Cui *and others*, 2017), their very general and flexible nature results in a lack of interpretability as well as a tendency to overfit the data (Zhang *and others*, 2008). The main reasons are: (i) the derived indices include all predictors $\boldsymbol{X}$, hence mixing very different variables for which a linear combination makes little sense, (ii) the very general vectors $\boldsymbol\alpha_j^T$ do not guarantee interpretability, and (iii) the flexibility of functions $g_j$ may result in complex functions preventing a clear interpretation of the corresponding index $Z_j$.

Usually, the predictors at hand can naturally be grouped into variables representing phenomena that jointly impact the response $Y$. For instance, grouped variables can be naturally interacting variables such as several weather, air pollutants, sociodemographic variables as well as lagged variables (Xia and Tong, 2006). Several authors proposed to take advantage of such groupings as a path to improve the interpretability of the derived indices (Li *and others*, 2010; Guo *and others*, 2015). This leads to the groupwise additive index model (GAIM) expressed as:


(1) \begin{align*}\label{eq1}
Y = \sum_{j = 1}^p g_j (\boldsymbol\alpha_j^T \boldsymbol{X}_j ) + \varepsilon,
\end{align*}

where the $\boldsymbol{X}_j \in \mathbb{R}^{l_j } (j = 1,\ldots,p)$ are subsets of variables of $\boldsymbol{X}$, i.e., $l_j < d$. The GAIM in (1) allows deriving more meaningful indices $Z_j = \boldsymbol\alpha_j^T \boldsymbol{X}_j$ since they are built from subsets of predictors that logically or naturally belong together. It can be seen as a sparser model since only a subset of variables enters a term in (1), noting that sparsity is a key aspect of interpretability (Rudin, 2019).

Although the GAIM in (1) allows an improvement in the indices interpretability, its flexibility can still result in physically or practically incoherent indices. Thus, it is also of interest to be able to constraint the indices weights $\boldsymbol\alpha_j$ to yield more meaningful indices. Constraints on the weights $\boldsymbol\alpha_j$ can represent additional information included in the model and reflect the expertise or knowledge specific to a given application context or operational requirements for the created indices. For an index to be useful in practice, it is also highly desirable that it relates to the response $Y$ in an easy-to-interpret way. For an air quality index, it is reasonable to expect $g_j$ to be monotonically increasing. Similarly, a temperature-related index may impose a convexity constraint on $g_j$, acknowledging a minimum mortality temperature and increased risks on both sides. A too flexible model for the function $g_j$ might however give implausible or difficult to interpret indices and therefore limit their usefulness for decision-making. This means that it is also of interest to impose constraints on the shape of the functions $g_j$.

In the present article, we propose a constrained groupwise additive index model (CGAIM) as a general model that includes all the constraints discussed above. It is a model of the form (1) in which constraints are added on the weights $\boldsymbol\alpha_j$ as well as on the functions $g_j$ depending on the application. Several authors proposed unconstrained GAIMs based on local linear estimation (Li *and others*, 2010; Wang *and others*, 2015; Guo *and others*, 2015; Wang and Lin, 2017). Fawzi *and others* (2016) proposed the addition of a few constraints on the weights $\boldsymbol\alpha_j$ but not on the functions $g_j$. Chen and Samworth (2015) proposed a PPR with shape-constrained functions $g_j$, but it is not in a groupwise context. Xia and Tong (2006) and then Kong *and others* (2010) proposed a GAIM with constraints on both the weights $\alpha_j$ and functions $g_j$, but limited to monotonicity and without the possibility to add additional covariates such as confounders. Finally, these methods all lack inference procedures to provide uncertainty assessment or test for specific indices of covariates. Such inference results are important for interpretation purposes. We propose here a general model that encompasses all mentioned ones, with the addition of an efficient estimation procedure, as well as index selection and inference.

## 2. The CGAIM

In order to present the proposed CGAIM, we rewrite and extend model (1) as:


(2) \begin{align*}\label{eq2}
Y = \beta_0 + \sum_{j = 1}^p \beta_j g_j (\boldsymbol\alpha_j^T \boldsymbol{X}_j ) + \sum_{k = 1}^d \gamma_k f_k
(W_k) + \boldsymbol{U}^T\boldsymbol\theta + \varepsilon,
\end{align*}

where $\boldsymbol{X}_j \in \mathbb{R}^{l_j } (j = 1,\ldots,p)$ are subsets of all the variables in $\boldsymbol{X}$, $\boldsymbol\alpha_j$ is a vector of weights and $g_j$ is a nonlinear function. The coefficients $\beta_j$ represent the relative importance of each index $Z_j = \boldsymbol\alpha_j^T \boldsymbol{X}_j$ in predicting the response $Y$. The constant $\beta_0$ is the intercept of the model.

The $W_k$ and $U$ (with dimension $\ge 1)$ represent additional covariates that are related to $Y$ but not entering any index. The formers are nonlinearly related to $Y$ through $f_k$ with importance $\gamma_k$, which are respective counterparts to $g_j$ and $\beta_j$, and the latter are linear. The typical example is confounding variables in environmental epidemiology such as day-of-week or time covariates.

One of the key features of the proposed CGAIM is to allow for any linear constraint on the weights $\boldsymbol\alpha_j$, i.e., constraints of the form $\boldsymbol{C}_j \boldsymbol\alpha_j \ge 0,$ where $\boldsymbol{C}_j$ is a $m_j \times l_j$ matrix, $m_j$ being the number of constraints and $l_j$ the number of variables in the group $\boldsymbol{X}_j$. Linear constraints allow for a large array of constraints. Examples include forcing some or all of the weights in $\boldsymbol\alpha_j$ being positive, in which case $\boldsymbol{C}_j$ is the identity matrix, and forcing them to be monotonically decreasing, in which case $\boldsymbol{C}_j$ is an $(l_j - 1)\times l_j$ matrix where $C_{j,pq} = 1$ when $p = q, C_{j,pq} = - 1$ when $p = q - 1$ and $0$ otherwise.

The other key feature of the CGAIM is the possibility to add shape constraints on the functions $g_j$ and $f_k$. Shape constraints include monotonicity, convexity, concavity, and combinations of the former (Pya and Wood, 2015). Note that not all functions $g_j$ and $f_k$ need to be constrained or have the same shape constraint.

For identifiability, we assume that the grouping is chosen before model fitting and that no predictor variable enters two indices, i.e., $\boldsymbol{X}_j \cap \boldsymbol{X}_k = \emptyset, \forall j,k$. Regarding the weights $\boldsymbol\alpha_j$, identifiability can be ensured by the classical unit norm constraint $\|\boldsymbol\alpha_j\| = 1$ with the first element of $\boldsymbol\alpha_j$ being positive (Yu and Ruppert, 2002; Yuan, 2011). However, we can also take advantage of linear constraints to ensure both identifiability and a better interpretability of the resulting indices. For instance, the constraints $\sum_{k = 1}^{l_j } \boldsymbol\alpha_{jk} = 1$ and $\boldsymbol\alpha_{j1} \ge 0$, which represents a weighted average of the variables in $\boldsymbol{X}_j$, are enough to ensure identifiability of $\boldsymbol\alpha_j$. As estimation of $g_j$s, $f_k$ and $\theta$ for fixed $\boldsymbol\alpha_j$ is a generalized additive model (GAM), we consider the classical centering identifiability constraints (Wood, 2004; Yuan, 2011). Finally, since we allow linear covariates in the model, we assume that no function $g_j$ is linear since it could cause identifiability issues in the groupwise context (a formal proof is provided by Fawzi *and others*, 2016).

## 3. Estimating the CGAIM

In this section, we present an estimation algorithm for the CGAIM based on the general framework of SQP. We first focus on the additive index part of the model for clarity purposes and then extend the estimation to the full model in (2). We also present a generalized cross-validation (GCV) criterion for model selection and two inference procedures.

### 3.1. Estimation problem

To fit the CGAIM, given observed data $(y_i,x_{i1},\ldots,x_{id})$, where $i = 1,\ldots,n$ and the $d$ predictor variables are partitioned into $p$ groups, we seek to minimize the squared error over coefficients $\beta_0$ and $\beta_j$, functions $g_j$ and weight vectors $\boldsymbol\alpha_j$, $j = 1,\ldots,p$, i.e.:


(3) \begin{align*}\label{eq3}
\begin{array}{cl}
\min& \sum_{i = 1}^n \left[ y_i - \beta_0 - \sum_{j = 1}^p \beta_j g_j (\boldsymbol\alpha_j^T \boldsymbol{x}_{ij})\right]^2\\
\mbox{subject} \ \mbox{to}& \boldsymbol{C}\boldsymbol\alpha \ge 0\\
&g_j \in m\\
\end{array},
\end{align*}

where $\boldsymbol\alpha = [\boldsymbol\alpha_1^T,\ldots,\boldsymbol\alpha_p^T]^T$ and $m$ is one of the shape constraints available for $g_j$.

Since the $\boldsymbol\alpha_j$s do not enter linearly in the squared error (3), this is a nonlinear least squares problem which suggests an approach such as a Gauss–Newton algorithm. However, an additional difficulty arises from the constraints of the model, especially those on the $\boldsymbol\alpha_j$s. It is thus appropriate to consider SQP steps, a general algorithm for nonlinear constrained optimization problems (Boggs and Tolle, 1995). It has been shown to work well in the context of nonlinear least squares (Schittkowski, 1988).

The proposed estimation methods for related models listed in the introduction (Xia and Tong, 2006; Li *and others*, 2010; Wang *and others*, 2015) are all based on local regression to minimize the sum of squares (3). However, it can be computationally intensive and makes the inclusion of constraints more difficult due to the high number of local coefficients to estimate. Here, we rather choose an approach based on splines for the function $g_j$ and SQP iterations for the weights $\boldsymbol\alpha_j$. Note that smoothing splines were shown to have good performances in a PPR context (Roosen and Hastie, 1994).

### 3.2. Estimation algorithm

Since the minimization problem (3) is a separable one (Golub and Pereyra, 2003), we propose here to estimate the GAIM with an algorithm that iteratively updates the functions $g_j$ and the weight vectors $\boldsymbol\alpha_j$. In the first step, with the $\boldsymbol\alpha_j$s fixed, we can derive indices values $z_{ij} = \boldsymbol\alpha_j^T \boldsymbol{x}_{ij}$. Estimating the functions $g_j$ is thus equivalent to estimating a GAM (Hastie and Tibshirani, 1986) using the current $z_{ij}$ as predictors. In such a model, $g_j$ can be efficiently estimated by smoothing splines as detailed by Wood (2017). After estimating the functions $g_j$, they are scaled to have norm one, and the coefficients $\beta_j$ are adjusted accordingly.

When shape constraints are considered, different corresponding methods can be considered. Pya and Wood (2015) proposed the shape-constrained additive models (SCAM) that estimates reparametrized P-spline coefficients through an iterative reweighted least squares like algorithm. Meyer (2018) proposed a constrained GAM (CGAM) that uses integrated and convex splines (Ramsay, 1988; Meyer, 2008) with quadratic programming to enforce shape constraints. Finally, Chen and Samworth (2015) proposed the shape-constrained additive regression that estimates nonsmooth shape-constrained functions through maximum likelihood. All these methods allow for monotonicity, convexity, and concavity constraints. Throughout the present article, we consider SCAM as it allows for a more flexible management of functions $g_j$ smoothness.

In the second step, with the functions $g_j$ estimated, we can update the weights $\boldsymbol\alpha_j$ by minimizing the sum of squares function (3) over the $\boldsymbol\alpha_j$ only. Let $\boldsymbol\alpha^{\text{old}}$ be the current value of $\boldsymbol\alpha = [\boldsymbol\alpha_1^T,\ldots,\boldsymbol\alpha_p^T]^T$ and $\boldsymbol\alpha^{\text{new}}$ the next value to be computed. The update $\delta = \boldsymbol\alpha^{\text{new}} - \boldsymbol\alpha^{\text{old}}$ can be conveniently computed by a quadratic program (QP) of the form


(4) \begin{align*}\label{eq4}
\begin{array}{cl}
\min&\boldsymbol\delta^T\boldsymbol{V}^T\boldsymbol{V}\boldsymbol\delta - 2\boldsymbol{V}^T\boldsymbol{R}\boldsymbol\delta\\
\mbox{subject} \ \mbox{to}& \boldsymbol{C}\boldsymbol\delta + \boldsymbol{C}\boldsymbol\alpha^{\text{old}} \ge 0\\
\end{array}
\end{align*}

in which $\boldsymbol{V}$ is the matrix containing the partial derivative according to the $\boldsymbol\alpha_j$ of the CGAIM equation, i.e., the right-hand side of (2). $\boldsymbol{V}$ contains $[\boldsymbol{v}_{i1},\ldots,\boldsymbol{v}_{ip}]$ at line $i$ with the vector $\boldsymbol{v}_{ij} = \boldsymbol{x}_{ij} \beta_j g_j^{\prime} (z_{ij} )$. $\boldsymbol{R}$ is the current residual vector that contains $r_i = y_i - \beta_0 - \sum_{j = 1}^p \beta_j g_j (\boldsymbol\alpha_j^{\text{old}^{T}}x_{ij})$. The objective function in (4) is a quasi-Newton step in which the Hessian part that involves the second derivatives of the CGAIM has been discarded to avoid its computational burden, leaving only the term $\boldsymbol{V}^T\boldsymbol{V}$. Thus, the update $\boldsymbol\delta$ is guaranteed to be in a descent direction. Discarding the second derivative of the model is a distinctive feature of least squares since it is usually negligible compared to the term $\boldsymbol{V}^T\boldsymbol{V}$ (Hansen *and others*, 2013). Note that this is especially true here since both the use of smoothing spline and shape constraints for $g_j (\cdot)$ results in smooth functions and thus low second derivatives $g_j^{\prime\prime} (\cdot)$. Finally, the constraints in (4) ensure that the updated vector $\boldsymbol\alpha^{\text{new}} = \boldsymbol\alpha^{\text{old}} + \boldsymbol\delta$ is still in the feasible region. Note that without these constraints, the problem in (4) reduces to a classical Gauss–Newton step for nonlinear least squares (Bates and Watts, 1988).

The algorithm alternates updating the weights $\boldsymbol\alpha_j$ and estimating the functions $g_j$ with the current $\boldsymbol\alpha_j$ until convergence. Convergence is usually reached when the least squares function (3) does not evolve anymore after updating the $g_j$ and $\boldsymbol\alpha_j$. Note that we can also consider other criteria for convergence such as stopping when the update $\delta$ is very small or the orthogonality convergence criterion of Bates and Watts (1981).

To start the algorithm, a constrained linear regression of the $[\boldsymbol{x}_{i1},\ldots,\boldsymbol{x}_{ip}]$ on $y_i$ should provide an initial guess $\boldsymbol\alpha_0 = [\boldsymbol\alpha_1^T,\ldots,\boldsymbol\alpha_p^T]$ close to the optimal solution (Wang *and others*, 2015). This constrained linear regression can be implemented as a QP of the form (4) by replacing $\boldsymbol{V}$ with the design matrix $\boldsymbol{X}$, and $\boldsymbol{R}$ with the response vector $\boldsymbol{Y}$. Alternatively, $\boldsymbol\alpha_0$ can be initiated randomly, using constrained random number generators (Van den Meersche *and others*, 2009). Key steps of the estimation procedure are summarized in Algorithm 1.

Algorithm 1 Constrained GAIM estimation

0. Initialize $\boldsymbol\alpha = [\boldsymbol\alpha_1^T,\ldots,\boldsymbol\alpha_p^T]$ either by a QP as in (4) or randomly.
1. Functions $g_j$ update:
  a. Estimate the $g_j$ by a SCAM with $y_i$ as the response and the $z_{ij} = \boldsymbol\alpha_j^T \boldsymbol{x}_{ij}$ as predictor.
  b. Scale the estimated $g_j$ to have unit norm and adjust the coefficients $\beta_j$ consequently.
2. Weights $\boldsymbol\alpha_j$ update:
  a. Compute the update $\boldsymbol\delta = [\delta_1,\ldots, \delta_p]$ through the QP (4).
  b. Set $\boldsymbol\alpha = \boldsymbol\alpha + \boldsymbol\delta$.
  c. Scale each $\boldsymbol\alpha_j$ to have unit norm.
3. Iterate steps 1 and 2 until convergence.

### 3.3. Additional covariates

The integration of the covariates $W_k$ and $U$ in the estimation procedure is straightforward since they only intervene in the update of functions $g_j$ (Step 1 of Algorithm 1). In this step, they are simply added as covariates in the SCAM (or GAM in the unconstrained case), along the current indices $Z_j$. Shape constraints can be applied on the functions $f_k$ as well. These terms do not intervene in the $\boldsymbol\alpha_j$ update step, since they are considered constants with respect to $\boldsymbol\alpha_j$, which mean that they disappear from the derivative matrix $\boldsymbol{V}$. Finally, note that the coefficients $\gamma_k$ are obtained as the norm of functions $f_j$.

### 3.4. Model selection

As the number of indices and covariates grow in the model, it is of interest to select a subset that are the most predictive of the response $Y$. To this end, we propose here a GCV type criterion of the form (Golub *and others*, 1979):


(5) \begin{align*}\label{eq5}
\mbox{GCV} = \frac{\sum_{i = 1}^n \left( {y_i - \widehat{y_i }}
\right)^2 / n}{\left( {1 - \text{edf} / n} \right)^2},
\end{align*}

where the numerator represents the residual error with $\widehat{y_i }$ the fitted value from the CGAIM, and the denominator is a penalization that depends on the effective degrees of freedom (edf). For a large number of indices $p$, we can perform the selection as a forward stepwise algorithm in which, at each step, the index minimizing the GCV is added to the model.

When a model can be reformulated linearly, the edf term in (5) can be estimated as the trace of the hat matrix, but it is not the case here. Instead, we consider a similar approximation as proposed by Roosen and Hastie (1994) for PPR, i.e.,


(6) \begin{align*}\label{eq6}
\mbox{edf} = p + d + \sum \left(\mbox{edf}_g \right) +
\sum\left(\mbox{edf}_{\alpha}\right),
\end{align*}

where $p + d$ charge one degree of freedom per index and covariate for the coefficients $\beta_j$ and $\gamma_k, \sum \left(\mbox{edf}_g\right)$ represent the sum of edfs for each ridge function smoothing, and $\sum\left( \mbox{edf}_{\boldsymbol\alpha} \right)$ is the sum of edfs for each index weight vector estimation. Estimation of $\mbox{edf}_g$ is well described in Meyer and Woodroofe (2000) and corresponds to the number of basic functions used in the smooth, to which we subtract the number of active constraints multiplied by a constant usually specified at $c \approx 1.5$ to account for the smoothing penalization (see also Meyer, 2018). Similarly, $\mbox{edf}_{\boldsymbol\alpha}$ can be estimated as the number of coefficients to which we subtract the number of active constraints (Zhou and Lange, 2013).

### 3.5. Inference

Inference for the ridge functions $g_j$ is well described elsewhere (Pya and Wood, 2015; Meyer, 2008) and inference for the coefficients $\beta_j$ is straightforward as they can be treated like regular regression coefficients using the $g_j ( \boldsymbol\alpha_j^T \boldsymbol{x}_{ij})$ as predictors. Here, we describe inference for the vector of weights $\boldsymbol\alpha = [\boldsymbol\alpha_1^T,\ldots,\boldsymbol\alpha_p^T]^T$ only. If one assumes normality of the residuals, then the transformed vector $\boldsymbol\xi = \boldsymbol{C}(\boldsymbol\alpha - \widehat{\boldsymbol\alpha})$ follows a truncated multivariate normal with null mean, covariance matrix $\boldsymbol{C}\boldsymbol\Sigma_{\boldsymbol\alpha} \boldsymbol{C}^T$, where $\boldsymbol\Sigma_{\boldsymbol\alpha}$ is the covariance matrix of $\boldsymbol\alpha$ for an unconstrained model, and lower bound $\boldsymbol{C}\left(\boldsymbol{b} - \widehat{\boldsymbol\alpha}\right)$ (Geweke, 1996). We can efficiently simulate a large number of vectors $\boldsymbol\xi^{\ast}$ from this truncated multivariate normal, and back-transform them as $\boldsymbol\alpha^{\ast} = \widehat{\boldsymbol\alpha}+\boldsymbol{C}^{-1}\boldsymbol\xi^{\ast}$ to obtain an estimate of the distribution of the vector $\boldsymbol\alpha$ (Botev, 2017). Empirical confidence intervals or other inference can then be obtained from the simulated $\boldsymbol\alpha^{\ast}$.

The unconstrained covariance matrix ${\boldsymbol\Sigma}_{\boldsymbol\alpha}$ can be obtained through the classical nonlinear least squares approximation ${\boldsymbol\Sigma}_{\boldsymbol\alpha} = s^2\left(\boldsymbol{V}^T\boldsymbol{V}\right)^{-1}$, where $s^2$ is an estimate of the residual variance of the model (Bates and Watts, 1988). In this instance, $s^2$ should be estimated using the effective degrees of freedom formula devised in Section 3.4. Note also that since it needs to be inverted, the constraint matrix $\boldsymbol{C}$ should be a square matrix. If this is not the case, it can be augmented by a matrix $\boldsymbol{C}_0$ spanning the row null space of $\boldsymbol{C}$ while the vector $\boldsymbol{b}$ is augmented with $-\infty$ (Tallis, 1965).

Without the normality assumption, inference and confidence intervals can be obtained through a bootstrap procedure (DiCiccio and Efron, 1996), with the following procedure. We start by extracting the residuals $\hat{\varepsilon}_i$ of the CGAIM fit. We then draw from the $\hat{\varepsilon}_i$ with replacement to obtain a new sample $\varepsilon_i^{\ast}$ that is then added to the fitted values to obtain a new response vector $y_i^{\ast} = \hat{y}+\varepsilon_i^{\ast}$ on which the CGAIM can be fitted (Efron and Tibshirani, 1993). We repeat this a large number $B$ of times to obtain a bootstrap distribution of any parameter from the CGAIM, including the weights $\boldsymbol\alpha_j$, the ridge functions $g_j$ and the coefficients $\beta_j$.

## 4. Simulation study

In this section, we analyze the performances of the CGAIM on different types of simulated data. We test the ability of the proposed CGAIM to estimate accurately weights $\boldsymbol\alpha_j$, by comparing it with other methods, its ability to find the most relevant predictors in the context of an important number of exposures, the ability of the GCV criterion to find the correct model and the coverage of the confidence intervals applied as described above.

### 4.1. Index estimation

In this setting, three predictor matrices are generated following a multivariate normal distribution of dimension $p_j = 4$ ($j = 1,2,3)$, with null means and covariance matrices having unit diagonal and nondiagonal elements equal to a predefined $\rho$ value. The first index is composed of sharply decreasing weights with a log function $g_1$ to emulate the effect of air pollution on mortality. The second includes moving average weights with a sigmoid function $g_2$ that represent a soft threshold on the index typical of logistic models. The third index represents a classical mortality temperature relationship with weights representing a delayed impact and a U-shaped relationship. The linear predictor is then the sum of the three ridge functions, i.e., with magnitudes $\beta_j = 1$ for $j = 1,2,3$ and an intercept $\beta_0 = 5$. A large number $n_s = 1000$ data sets are generated by adding Gaussian white noise to the linear predictor described here. More details on the simulation setup are given in Supplementary material available at *Biostatistics* online.

From the basic mechanism described above, various scenarios are implemented. In these scenarios, we change the sample size of simulated data with $n = 100, 200, 500, 1000$, the correlation between predictor variables with nondiagonal elements of the covariance matrix in $\rho = 0, 0.25, 0.50, 0.75$, and the noise level with standard deviations in $\sigma = 0.2, 0.5, 1$. The unconstrained GAIM and the CGAIM are applied on each of the generated data sets. The CGAIM is applied with the constraints that all weights are positives that $\boldsymbol\alpha_1$ is increasing and $\boldsymbol\alpha_2$ decreasing. The functions $g_1$ and $g_2$ are constrained to be both monotonically increasing and $g_3$ is constrained to be convex. The specific constraints applied to each index are summarized in Table S1 of the Supplementary material available at *Biostatistics* online. The GAIM is only applied with identifiability constraints, i.e., that non-negativity of the first element of each weight vector $\boldsymbol\alpha_{j1} \ge 0$ and unit norm for $\boldsymbol\alpha_j$. To test the model with wrongly specified constraints, we fit a mis-specified model (MGAIM) constraining $\boldsymbol\alpha_1$ to be decreasing and $\boldsymbol\alpha_2$ increasing. For CGAIM and MGAIM, we fix the smoothness of $g_j$ to an equivalent of 10 degrees of freedom. This avoids the computational burden of smoothness optimization in SCAM, while keeping enough flexibility for model fitting.

Besides the three models described above, three benchmark models are applied on the generated data sets. The first one is the PPR as the most general additive index model available. Comparing the (unconstrained) GAIM to the PPR allows assessing the benefits of defining groups of variables *a priori*. The second benchmark is the groupwise minimum average variance estimator (gMAVE) of Li *and others* (2010), as representative of groupwise dimension reduction methods. It allows the evaluation of the estimation method without constraint. Finally, we also apply the functional additive cumulative time series (FACTS) model of Kong *and others* (2010), that contains a groupwise additive structure and monotonicity constraints on both index weights $\boldsymbol\alpha_j$ and ridge functions $g_j$. We only apply the monotonicity constraints applied to CGAIM to FACTS, as its extension to other type of constraints in not trivial. The performances are evaluated by comparing the estimated weights $\widehat{\boldsymbol\alpha}_j$ to the true values $\boldsymbol\alpha_j$. The quality of estimated $\widehat{\boldsymbol\alpha}_j$ are evaluated using the classical root mean squared errors (RMSE) that aggregates information about both the bias and standard error of the estimators.

**Fig. 1.**
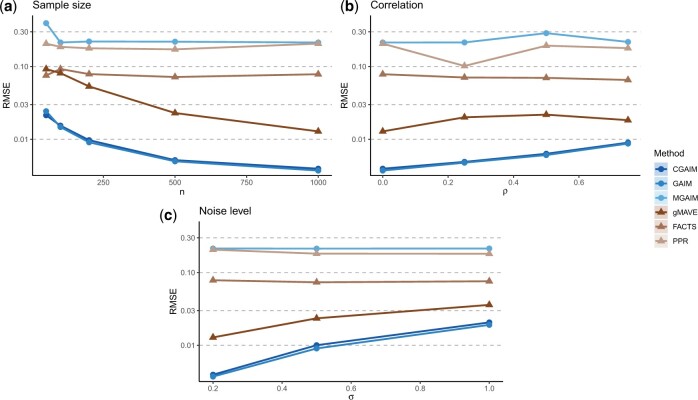
Estimated RMSE for different scenarios, varying the sample size (a), the correlation between variables (b), and the noise level (c). Note that the CGAIM and GAIM curves overlap each other at the bottom. Note the log scale.

Figure 1 shows the RMSE for each model for different sample sizes, correlation coefficient between the predictor variables and noise levels. There is overall a clear hierarchy between the compared models, with the GAIM and CGAIM having the lowest errors, the gMAVE having slightly higher errors and being more sensitive to the sample size, and then the FACTS. PPR have overall much higher errors being in addition extremely variable. The methods based on the proposed algorithm on the other hand show important stability with robustness to variation in all explored parameters. As expected, the MGAIM shows low performances because of the mis-specified constraints preventing the model to converge to the true $\boldsymbol\alpha_j$. Note however that it displays very low variance, as it converges towards the best solution within the feasible region (see Supplementary material available at *Biostatistics* online).

### 4.2. Index selection

In this experiment, we evaluate the ability of the GCV criterion (5) to retrieve the correct model. We consider the structure detailed in the previous experiment with $n = 1000$ and $\rho = 0$, as well as three noise levels $\sigma = 0.2, 0.5,$ and $1$. For each realization, we randomly select $p^{\ast}$ indices $J^{\ast} \subset \{1,2,3\}$ and attribute them a unit coefficient $\beta_{j \in J^{\ast} } = 1$. We attribute $\beta_{j \notin J^{\ast} } = 0$ to the remaining indices, thus discarding them from the generated model. At each realization, we choose the best model by GCV and compute the sensitivity and specificity. Sensitivity is defined as the proportion of indices in $j^{\ast}$ that are in the model selected by GCV, and specificity the proportion of indices not in $j^{\ast}$ that are discarded by the GCV.

**Fig. 2.**
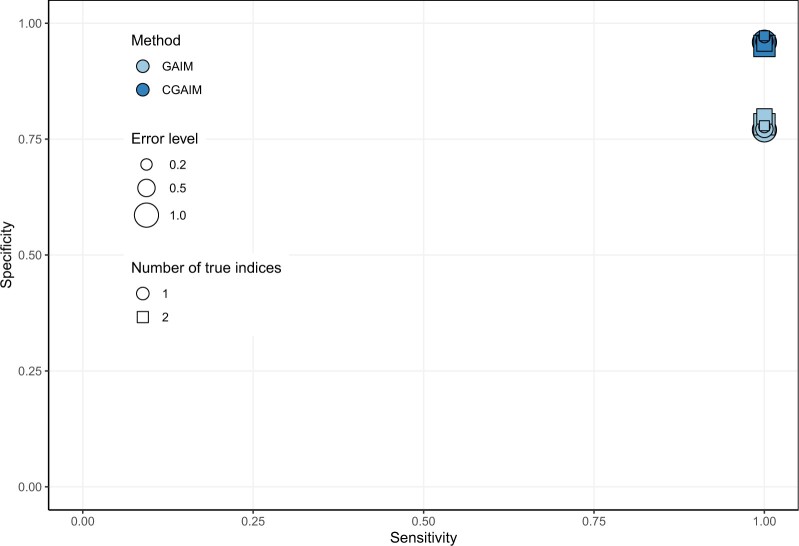
Average sensitivity and specificity of index selection computed on the 1000 simulations for various number of true indices and error level.

Figure 2 shows the average sensitivity and specificity on $n_s = 1000$ realizations for the two number of non-null indices and various noise levels. Sensitivity is equal to one in all simulations, meaning that the GCV always selects the true indices in the model. Specificity indicates that the GCV select only the true indices most of the time, i.e., around 95$\%$ of the time for the CGAIM and around 80$\%$ of the time for GAIM. The GAIM might then be prone to slight overfitting, while the constraints in the CGAIM allow achieving more parsimonious models. However, specificity is still high in all cases and is not sensitive to the noise level. The proposed GCV criterion is therefore mostly successful for model selection.

### 4.3. Coverage

To evaluate the inference procedures proposed for the CGAIM, we perform simulations to assess the coverage achieved by confidence intervals for the $\boldsymbol\alpha_j$ weights. We generate data sets following the same mechanism described above, with $n = 1000$ and $\rho = 0$, as well as three noise levels $\sigma = 0.2, 0.5,$ and $1$. We then fit a CGAIM model as in the two first experiments and estimate its 95$\%$ confidence interval using both the normal approximation and residual bootstrap. In both cases, the number of created samples is fixed to $B = 500$. As the constraints can create some bias, especially for coefficients involved in active constraints, we compute the bias-corrected coverage, as the proportion of confidence intervals containing the average estimated values $\widehat{\alpha}_j$ from the simulations (Morris *and others*, 2019).

**Fig. 3.**
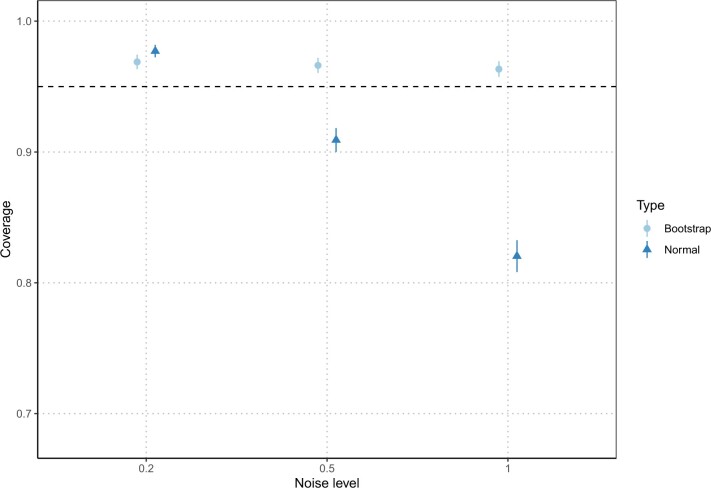
Estimated coverage for both inference methods and various noise level. Vertical segments indicate $\pm$1 standard error of the coverage.

Figure 3 shows the bias-corrected coverage for both method and the three noise levels. The residual bootstrap shows constant reasonable coverage with values around 96$\%$ for all noise levels. In contrast, the normal approximation method is widely affected by the noise level and shows important coverage errors, with major underestimation for the highest noise level. This coverage error can also significantly vary between the various $\boldsymbol\alpha_j$ with low coverages for $\boldsymbol\alpha_3$ specifically, while the variation between indices is lesser for the residual bootstrap (see Figure S4 of the Supplementary material available at *Biostatistics* online).

### 4.4. Exposome

In this experiment, we depart from the structure of the previous experiments and apply the GAIM and CGAIM to estimate the most important predictors in a simulation study typical of exposome studies. We modify the simulation study proposed by Agier *and others* (2016), using the structure of the HELIX cohort (Robinson *and others*, 2018). We generate a matrix of $d = 28$ predictors with $n = 1200$ subjects from the correlation matrix provided in Robinson *and others* (2018). In each realization, we select $p^{\ast} = 5,10,15$ predictors $K^{\ast} \subset \left\{ {1,\ldots,28} \right\}$ to have non-null weights, while the remaining predictors are attributed null weights, and have therefore no association with the response. We then generate the response vector through the model $Y^{\ast} = \sum_{k \in K^{\ast} } \alpha_k X_k + \varepsilon,$ where $\boldsymbol\alpha_k$ is either -1 or 1 with equal probability to evaluate the ability of the model estimate the direction of the association. Response vectors are then generated such that the $R^2$ of the model is $3p^{\ast} / 100$ (Agier *and others*, 2016).

As the correlation matrix used to generate the predictors $X_k$ represents environmental stressors, five groups naturally arise (Nieuwenhuijsen *and others*, 2019): climatic ($l_1 = 4)$, air pollution ($l_2 = 5)$, traffic-related ($l_3 = 4)$, natural environment ($l_4 = 5)$, and built-environment ($l_5 = 10)$ variables. We apply the (unconstrained) GAIM and CGAIM on 1000 realizations of the above-described mechanism with these groups of variables. The CGAIM is applied with the constraints $| \boldsymbol\alpha_k | \le 1 \forall k \in \left\{ {1,\ldots,28} \right\}$, convexity constraint on $g_1$ (representing the effect of climate) and increasing monotonicity on other $g_j$ ($j \ne 1)$. We then compare the estimated $\widehat{\boldsymbol\alpha}_k$ ($k \in \left\{ {1,\ldots,28} \right\})$ to the true value $\boldsymbol\alpha_k$.

**Fig. 4.**
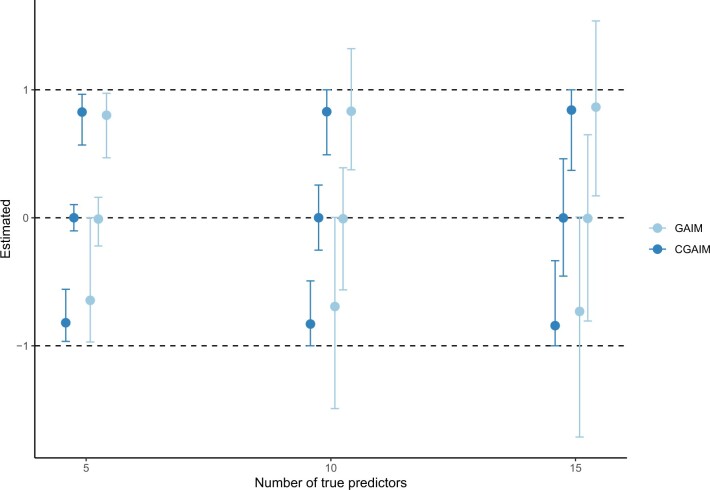
Average estimated $\widehat{\boldsymbol\alpha}_j$ for according to the true value $\boldsymbol\alpha_j$. Segments indicate 2.5th and 97.5th percentile of estimated $\boldsymbol\alpha_j$.

Figure 4 shows that the $\widehat{\boldsymbol\alpha}_k$ are on average close to the true $\boldsymbol\alpha_k$, successfully discriminating null weights but also the direction of non-null weights. They are closer to the true value for the CGAIM compared to GAIM, with also much lower variability in the estimated weights. The difference between estimated and true weights also slightly decreases with the number of non-null weights $\boldsymbol\alpha_k$. Therefore, the CGAIM, performs well with many predictors and complex correlation patterns, especially when constraints are considered.

## 5. Application

This section presents an example of application in environmental epidemiology in which the CGAIM is used to construct multiple indices representing heat-related mortality risks. A second application on air pollution is presented in Supplementary material available at *Biostatistics* online. This application considers daily mortality and exposure data spanning the months of June–August for the period 1990–2014 ($n = 2300)$ from the Metropolitan Area of Montreal in the province of Quebec, Canada, which are described in detail in, e.g., Masselot *and others* (2018, 2019). Briefly, daily all-cause mortality data are provided by the province of Quebec National Institute of Public Health, while daily temperature and humidity data are extracted from the 1 km $\times$ 1 km gridded data set DayMet (Thornton *and others*, 2021).

We apply the CGAIM to find optimal weights for temperature indices that represent potentially adverse effects. Indices created include lagged averages of $T\min$ and $T\max$, following the current indices in Montreal (Chebana *and others*, 2013), and we also include the vapor pressure ($Vp)$ variable to represent humidity, since it is also sometimes considered a determinant of summer mortality (e.g., Barreca, 2012). The objective here is to give an example of the possibilities offered by the CGAIM. Thus, to estimate these indices, the following full model is considered:


(7) \begin{align*}\label{eq7}
Y& = \beta_0 + \beta_1 g_1 (\boldsymbol\alpha_1^T T\min_{3d} ) + \beta_2 g_2
(\boldsymbol\alpha_2^T T\max_{3d} ) + \beta_3 g_3 (\boldsymbol\alpha_3^T Vp_{3d} )\nonumber\\
&\quad +\gamma_1 f_1 (\mbox{DOS}) +
\gamma_2 f_2 (\mbox{year}) +\varepsilon,
\end{align*}

where $Y$ is the all-cause daily mortality, $T\min$, $T\max_{3d}$, and $Vp_{3d}$ represent matrices of lags 0, 1, and 2 days of corresponding variables, meaning that the $\boldsymbol\alpha_j (j = 1,\ldots,3)$ are vectors of length 3. The two additional covariates are the day-of-season and year variables to control for the seasonality, interannual trend, and residual autocorrelation as commonly done in time series study in environmental epidemiology (Bhaskaran *and others*, 2013).

We consider a CGAIM and an (unconstrained) GAIM. The CGAIM model includes constraints for positive and decreasing weights with the lag, i.e., $\alpha_{j0} \ge \alpha_{j1} \ge \alpha_{j2} \ge 0, \forall j$. This is encoded by the following constraint matrix


(8) \begin{align*}\label{eq8}
C_j = \left[\begin{array}{ccc}
0& 0 & 1\\
1&-1& 0 \\
0 & 1 & -1 \\
\end{array}\right]\!.
\end{align*}

For the indices to directly represent a measure of heat risk, and because the data are restricted to the hottest months of the year with little exposure to cold, we add the constraint that the relationship $g_j$ is monotone increasing for all $j$. As in the simulation study, we fix the smoothness to the equivalent of 10 degrees of freedom. Confidence intervals are computed through the residual bootstrap.

For both the CGAIM and GAIM, we use the GCV criterion (5) to determine the best set of indices to predict summer mortality. Bets models include $T\max$ and $Vp$ for both the GAIM and CGAIM. Among these two best models, the GCV of the CGAIM one is slightly lower being at 90.2 compared to 90.6 for the GAIM.

**Fig. 5.**
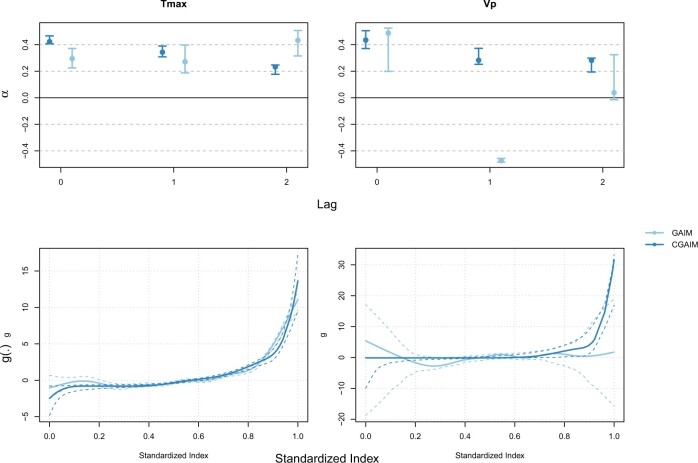
Resulting indices created in Montreal. Top row: weights $\boldsymbol\alpha_j$ for each selected index; bottom row: functions $\boldsymbol{g}_{\boldsymbol{j}}$. Indices have been standardized over the range [0–1] for ease of comparison. Each column corresponds to one index. Vertical segments and dotted lines represent block bootstrap 95$\%$ confidence intervals.

The indices and their association with mortality are shown in Figure 5. The CGAIM attributes a slightly decreasing weights with lags of $T\max$ resulting in an index that has a large association with mortality for extreme values, especially above the value 0.9 of the standardized index (around 32$^{\circ}$C). Note that this value is slightly below the current $T\max$ threshold in Montreal. The GAIM attributes a slightly larger weight to lag 2 of $T\max$ compared to lag 0 and 1, but with larger confidence intervals compared to the CGAIM. The $g$ curve is similar to the one of the CGAIM but less smooth. Regarding *Vp*, the results from the CGAIM is very similar to those of Tmax, the weights roughly spread across lags and with a relationship sharply increasing at highest values of the index. In contrast, the GAIM attributes two opposite weights for lag 0 and 1 of Vp and a null weight for lag 2 with a ridge function oscillating around the zero line. Given the flatness of the ridge function, such a contrast between GAIM or CGAIM could either suggest the influence of unmeasured confounding, or some overfitting from the models. Indeed, evidence regarding the role of humidity in heat-related mortality is overall weak and inconsistent (Armstrong *and others*, 2019).

## 6. Discussion

Following the growing need of understanding the impact of mixtures of environmental exposures on human health, the present article proposes a method to construct indices with constraints under the form of a CGAIM. The CGAIM is expected to be of use both for modeling and creating comprehensive indices for public health stakeholders. Its strengths include the possibility to include a high number of predictors $\boldsymbol{X}_j$ (including lags), include additional prior information from public health experts, and construct multiple indices simultaneously. Compared to previous work on the subject, the key novelties of the work are thus: (i) the possibility to add any linear constraints on the index weights $\boldsymbol\alpha_j$, (ii) the inclusion of constrained smoothing in the model to improve the indices usefulness, (iii) a simple and efficient algorithm to estimate the indices, and (iv) a criterion for index selection.

The constraints allow the proposed model to integrate additional information reflecting prior assumptions about the studied associations as well as integrate operational limitations to constructed indices. Examples of useful prior assumption include constraining indices and function shape to be convex for temperature-related mortality studies, or increasing for air pollution-related mortality studies, for which usual flexible methods may fail (Armstrong *and others*, 2020). Constraints can also force coefficients towards a specific feasible region to better control for unmeasured confounding causing issues such as the reversal paradox (Nickerson and Brown, 2019). Adding such constraint for prior information, if correctly specified, also results in quicker convergence as shown by the timings reported in Supplementary material available at *Biostatistics* online. On the other hand, operational constraints force constructed indices to have specific desirable properties. For instance, it is desirable that monitored heat indices reflect two constraints: (i) decreased influence of higher lags to account for increased uncertainty in weather forecasts and (ii) a monotonic association with mortality for ease of interpretation. Such constraints might be desirable even at the expense of more optimal solutions. Although most applications displayed in this article include non-negativity constraints, this is not a specificity of the method, and constraints with negative coefficients are possible, for instance to construct exposure representing differences between variables.

A simulation study shows that the CGAIM can accurately estimate the index weights as well as the index relationship with the response variable compared to other advanced and recent models which is a step further in obtaining representative indices for practical applications. It shows that constraints help the model recover the true coefficient values. The simulation study also shows the model is robust to low sample sizes, highly correlated predictors, low signal-to-noise ratio, and high dimension with complex correlation patterns. The CGAIM is also compared to the PPR to evaluate the benefits of grouping variables, to the gMAVE as well as FACTS algorithms. Comparisons suggest that the CGAIM is more stable than these algorithms. In fact, even without any constraint, the proposed algorithm is efficient and converges quickly to an optimal solution, as shown by the comparison between the GAIM and gMAVE (see Supplementary material available at *Biostatistics* online for a comparison of computational burden). In addition, simulation studies of Sections 4.2 and 4.3 show that the model can efficiently recover the indices and variables that are the most predictive of the response.

Another strength of the work is in proposing and evaluation two inference procedures, an aspect of multiple index models that is often neglected in multiple index models, except in recently proposed Bayesian methods (McGee *and others*, 2022). One proposed procedure is based on a normal approximation of constrained nonlinear least squares, and one based on bootstrap resampling. Both methods however display non-negligible coverage error for confidence intervals. The normal approximation can especially widely underestimate the uncertainty. This is mainly related to the covariance matrix constructed from nonlinear least squares that have been shown to significantly underestimate coverage even in far simpler settings (Donaldson and Schnabel, 1987). In contrast, bootstrap-based confidence intervals provide more satisfactory results although Section 4.3 shows that they tend to overestimate uncertainty which is also consistent with previous work on bootstrap confidence intervals (Carpenter and Bithell, 2000). Inference in constrained settings often presents mixed results (Meyer, 2018), and further work is necessary to improve this aspect of the method.

The proposed method assumes that the variables and their grouping is selected *a priori*, with the idea that in many cases, the researcher has a clear idea of the relevant variables to be included. This assumption is reasonable in many applications in which a natural grouping of variables arises. For instance, in environmental epidemiology, exposure variable can often be grouped into category such as climate, air pollution, or built-environment variables. Common tools to determine which variables to include in a study such as directed acyclic graphs (Greenland *and others*, 1999) or clustering (Song and Zhu, 2016) can also be used to determine a grouping *a priori*. When a limited number of concurrent groupings are investigated, the GCV criterion proposed in the present work can be used to decide. However, there may also be a need for a more automated selection procedure and an area for future research is thus to propose a flexible grouping mechanism. This is a difficult problem as the number of possible classifications increases dramatically with the number of variables.

Another limit of the proposed CGAIM is that it is currently restricted to continuous responses. Although this encompasses many situations, including counts when they are large enough such as in the applications above, it is of interest to extend this work to special cases such as logistic regression or survival analysis to increase its applicability. It is thus of interest to develop a generalized version of the CGAIM, in the same fashion as the generalized extension of the PPR (Roosen and Hastie, 1993; Lingj and Liestøl, 1998).

## Supplementary Material

kxac023_Supplementary_Data

## Supplementary material

Supplementary material is available at http://biostatistics.oxfordjournals.org. It includes full details on the data-generating mechanisms and additional results from the simulation study. A second application of the CGAIM to an air pollution index is also presented in Supplementary Materials. An R package cgaim implementing the method is freely available on Github and CRAN. The reproducible code for the simulations and applications is freely available on the first author’s GitHub (https://github.com/PierreMasselot/Paper–2022–Biostatistics–CGAIM).
