# Supplementary material for: Constrained groupwise additive index models
Source: Biostatistics. 2022 Jul 6;24(4):1066–84. doi: 10.1093/biostatistics/kxac023 (PMC10583725; doi:10.1093/biostatistics/kxac023)
Supplement: kxac023_Supplementary_Data [file kxac023_supplementary_data.docx]

Constrained groupwise additive index models

Supplementary materials

# Additional information on simulations

## Data-generating mechanism: experiments 1 to 3

In all experiments, we start by generating the predictor matrices $\boldsymbol{X}_{1}$, $\boldsymbol{X}_{2}$ and $\boldsymbol{X}_{3}$ all of dimension $n\times4$, meaning that the three indices to be constructed include 4 variables each. Each $\boldsymbol{X}_{j}$ is generated from a multivariate normal distribution $MVN\left( \boldsymbol{0},\Sigma\right)$ where the covariance matrix is fixed as:

|  | $\Sigma=\left( \begin{matrix} 1 & \rho& \rho& \rho\\ \rho& 1 & \rho& \rho\\ \rho& \rho& 1 & \rho\\ \rho& \rho& \rho& 1 \end{matrix} \right)$ | (1) |
| --- | --- | --- |

Once the matrices $\boldsymbol{X}_{j}$ are generated, true indices are created as $Z_{j}=\boldsymbol{X}_{j}\boldsymbol{\alpha}_{j}$, and are then scaled between 0 and 1 before being passed through ridge functions $g_{j}$. Evaluated ridge functions are then summed with the addition of an intercept $\beta_{0}=5$. Weights $\boldsymbol{\alpha}_{j}$ and functions $g_{j}$ are given in Supplementary Table 1 with the latter illustrated in Supplementary Figure 1. The data-generating model for the linear predictor $Y^{*}$ is then

|  | $Y^{*}=5+\sum_{j=1}^{3} \beta_{j}g_{j}(\boldsymbol{\alpha}_{j}^{T}\boldsymbol{X}_{j})$ | (2) |
| --- | --- | --- |

where $\beta_{j}=1 \forall j$.

Finally, a large number $n_{s}=1000$ error vectors are generated from a normal distribution with mean 0 and standard deviation $\sigma$. These are added to $Y^{*}$ to obtain the response vectors used in the simulations.

In the first experiment reported in the main manuscript, several scenarios are considered with various values for the sample size $n$, the correlation $\rho$ in Equation (1), and the error standard deviation $\sigma$. The full list of scenarios is found in Supplementary Table 2.

The second and third experiments (sections 4.2 and 4.3 of the main manuscript) only implement scenarios 1, 9 and 10 of Supplementary Table 2, *i.e.,* only varying the noise level in generated data. In the second experiment, $\beta_{j}$s in equation (1) are randomly set to 0 with two additional scenarios, one in which a single $\beta_{j}$ is non-null, and one in which two $\beta_{j}$s are non-null.

Supplementary Table 1: Design and CGAIM application for the simulation study.

|  | True model | | Applied constraints | |
| --- | --- | --- | --- | --- |
| Index | Weights $\alpha_{j}$ | Function $g_{j}\left( Z_{j} \right)$ | CGAIM | MGAIM |
| 1 | $\left. \left( 0.7, 0.2, 0.1, 0 \right. \right)^{T}$ | $e^{Z_{1}}$ | $\alpha_{11}\geq\alpha_{12}\geq\alpha_{13}$  $g_{1}$ increases monotonically | $\alpha_{11}\leq\alpha_{12}\leq\alpha_{13}$  $g_{1}$ increases monotonically |
| 2 | $\left. \left( 0, 0, 0.5, 0.5 \right. \right)^{T}$ | $\frac{1}{1+e^{-{5Z}_{2}}}$ | $\alpha_{21}\leq\alpha_{22}\leq\alpha_{23}$  $g_{2}$ increases monotonically | $\alpha_{21}\geq\alpha_{22}\geq\alpha_{23}$  $g_{2}$ increases monotonically |
| 3 | $\left. \left( 0.2, 0.4, 0.3, 0.1 \right. \right)^{T}$ | $\sum_{p=1}^{5} \delta Z_{3}^{p}$  $\delta=(0.212, 0.301, 0.098,$  ${0.015, 0.002)}^{T}$ | $g_{3}$ convex | $g_{3}$ concave |


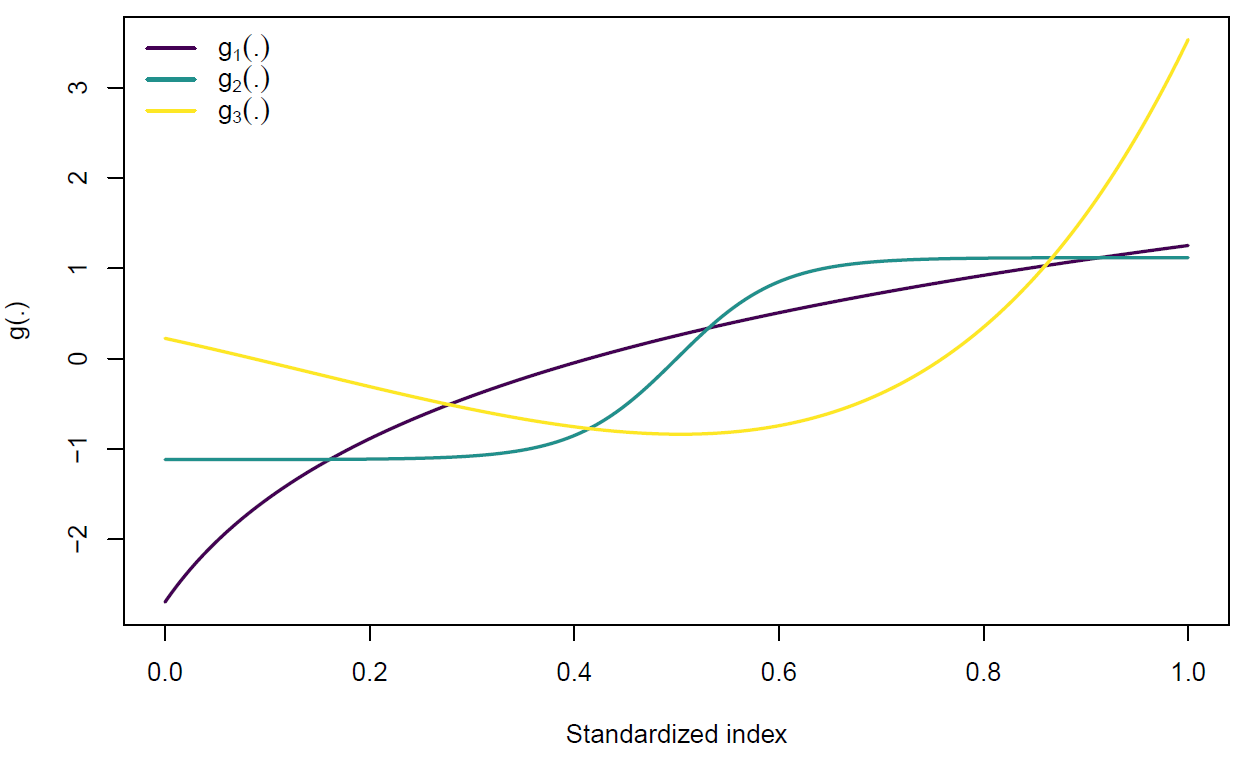


Supplementary Figure 1: Illustration of true ridge functions used in the data-generating mechanism. Equations of each function are found in Supplementary Table 1.

Supplementary Table 2: List of scenarios from section 4.1 of main manuscript. $n$: sample size; $\rho$: correlation between variables; $\sigma$: error standard deviation.

| Scenario | Experiment | $n$ | $\rho$ | $\sigma$ |
| --- | --- | --- | --- | --- |
| 1 | Baseline | 1000 | 0.00 | 0.2 |
| 2 | Varying $n$ | 50 | 0.00 | 0.2 |
| 3 |  | 100 |  |  |
| 4 |  | 200 |  |  |
| 5 |  | 500 |  |  |
| 6 | Varying $\rho$ | 1000 | 0.25 | 0.2 |
| 7 |  |  | 0.50 |  |
| 8 |  |  | 0.75 |  |
| 9 | Varying $\sigma$ | 1000 | 0.00 | 0.5 |
| 10 |  |  |  | 1.0 |

## Data-generating mechanism: experiment 4

In this experiment, we generate a predictor matrix $\boldsymbol{X}$ of size $1200\times28$ as a multivariate normal with null mean vector and correlation matrix extracted from Robinson *and others* (2018) shown in Supplementary Figure 2. For each realization, we create the linear predictor as $Y^{*}=\boldsymbol{X\beta}$ where each component in $\boldsymbol{\beta}$ is randomly drawn from $\{-1;0;1\}$. We consider three scenarios in which we fix the number of non-null components to $p^{*}=5,10,15$. Among the non-null coefficients, -1 and 1 are drawn with equal probability. Note that, as variables are grouped in the models (see below), we ensure at least one non-null coefficient in each group.

Once the linear predictor $Y^{*}$ is constructed, response vectors are generated by adding a random error from a gaussian distribution with null mean and variance $\sigma^{2}$. The variance is chosen such that the $R^{2}$ between the linear predictor and simulated response is roughly equal to $3p^{*}\%$. This can be achieved by setting the variance to

|  | $\sigma^{2}=Var\left( Y^{*} \right)\frac{R^{2}}{1-R^{2}}$ | (3) |
| --- | --- | --- |

On each realisation, we apply both the CGAIM and an (unconstrained) GAIM, in which the 28 variables are gathered into five groups. This classification is given in Supplementary Table 3 and shown in the correlation matrix of Supplementary Figure 2. The CGAIM is applied with the constraints that $\left| \alpha_{jk} \right|\leq{p_{j}^{*}}^{-1}$ where $p_{j}^{*}$ is the number of non-null coefficients in $\boldsymbol{\beta}$ that are in group $j$, in order to account for the unit norm identifiability constraint. Note that the absolute value constraint can be enforced as the double constraint $\alpha_{jk}\leq{p_{j}^{*}}^{-1}$ and $-\alpha_{jk}\leq{p_{j}^{*}}^{-1}$. In the end, estimated $\hat{\alpha}_{jk}$ are then post-multiplied by $p_{j}^{*}$ in order to compare them independently of the scenario on $p_{j}^{*}$. Finally, we also add increasing monotonicity constraints on all ridge functions $g_{j}$ except on the group representing weather variables for which the function $g_{j}$ is constrained to be convex.


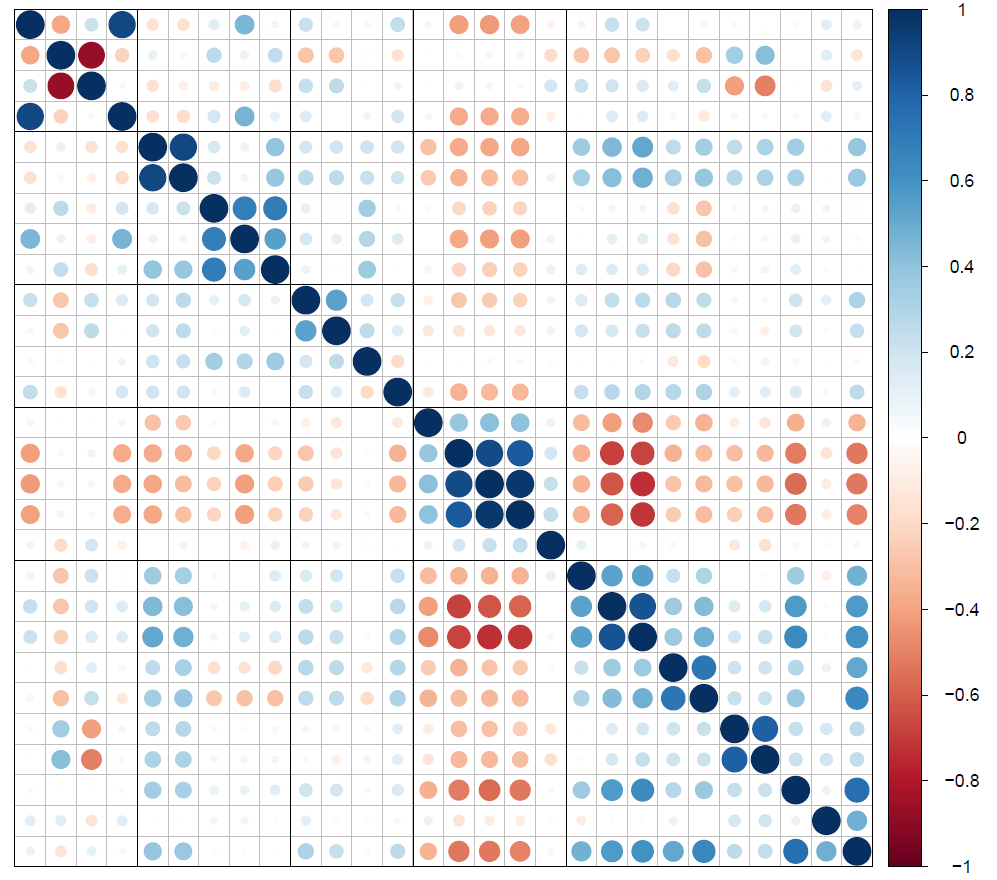


Supplementary Figure 2: Correlation matrix of the predictor matrix $\boldsymbol{X}$ in experiment 4. Black lines delimit groups applied in the CGAIM.

Supplementary Table 3: List of variables and groups represented in the correlation matrix extracted from Robinson *and others* (2018). The order of variables corresponds to the order of rows and columns in the correlation matrix of Supplementary Figure 2.

| **Group** | **Variable** | **CGAIM constraint** |
| --- | --- | --- |
| Meteorology | Temperature | Convex |
|  | Relative humidity |  |
|  | Atmospheric pressure |  |
|  | UV irradiance DNA damaging dose |  |
| Air pollution | NO_2_ | Monotone increasing |
|  | NO_x_ |  |
|  | PM_2.5_ |  |
|  | PM_10_ |  |
|  | PM absorbance |  |
| Road | Noise level | Monotone increasing |
|  | Traffic load of all road in 100 m radius |  |
|  | Traffic density on nearest road |  |
|  | Distance to nearest road |  |
| Natural environment | Distance to nearest large green space | Monotone increasing |
|  | NDVI within radius of 100m |  |
|  | NDVI within radius of 300m |  |
|  | NDVI within radius of 500m |  |
|  | Distance to nearest large blue space |  |
| Built environment | Population density | Monotone increasing |
|  | Building density within radius of 100m |  |
|  | Building density within radius of 300m |  |
|  | Connectivity density within radius of 100m |  |
|  | Connectivity density within radius of 300m |  |
|  | Number of bus stops within radius of 300m |  |
|  | Number of bus stops within radius of 500m |  |
|  | Facility richness within a radius of 300m |  |
|  | Land use SEI within radius of 300m |  |
|  | Walkability within radius of 300m |  |

## Implementation of models

Implementation of the CGAIM (including unconstrained and mis-specified models) is provided in the R package cgaim available on CRAN. The PPR is implemented through the function ppr available in the package stats in R. The gMAVE and FACTS have been fully implemented in R by the authors in order to be compared in simulations, with the code made available along the simulation and application code on Github.

As all methods rely on iterative fitting procedures, we used common algorithm control parameters, *i.e.*, with a tolerance of 0.001 for convergence of the least-squares criterion, and a maximum of 50 iterations when the algorithm does not converge. To match the identifiability constraints of the CGAIM and for fairer comparison, estimated ${\hat{\boldsymbol{\alpha}}}_{j}$ are normalized to unit norm for all models after fitting. The gMAVE and FACTS models also require a bandwidth that is set to the optimal bandwidth given in Li *and others* (2010). Finally, note that although only monotonically decreasing constraints are mentioned in the FACTS model for both index weights and ridge functions (Kong *and others* 2010), we added convexity and concavity constraints to the ridge functions. Due to the structure of the algorithm however, it was not straightforward to add additional constraints to the $\boldsymbol{\alpha}_{j}$ coefficients.

## Additional results

Supplementary Figure 3 shows distribution of the estimated ${\hat{\boldsymbol{\alpha}}}_{j}$ across simulations, providing more details on the RMSE reported in Figure 1 of the main manuscript. In particular, the GAIM and CGAIM show both low bias and low variance. As expected, the MGAIM displays high bias due to mis-specified constraints, but very low variance, even with important noise levels. This indicates that the algorithm consistently converges to the best solution within the specified constraints. In contrasts the gMAVE displays little bias but relatively higher variance than the CGAIM, while the FACTS also exhibits non-negligible bias. Finally, the PPR displays both important bias and variance, being an overly flexible method.

Supplementary Table 4 indicate average execution time of each model showing the low execution time of our Gauss-Newton based algorithm, compared to the expensive fully non-parametric Kernel-based approaches of FACTS and gMAVE. One can also note that, for similar RMSE, the CGAIM is overall less time consuming than the GAIM. Therefore, well specified constraints allow for quicker convergence.

Supplementary Figure 4 shows estimated bias-corrected coverage for each $\alpha_{jk}$. The residual bootstrap shows consistently over-estimated coverage apart for few instances for the highest noise level, although it remains close to the nominal coverage of 95%. On the other hand, coverage for the normal approximation widely varies, being grossly underestimated for the third index.


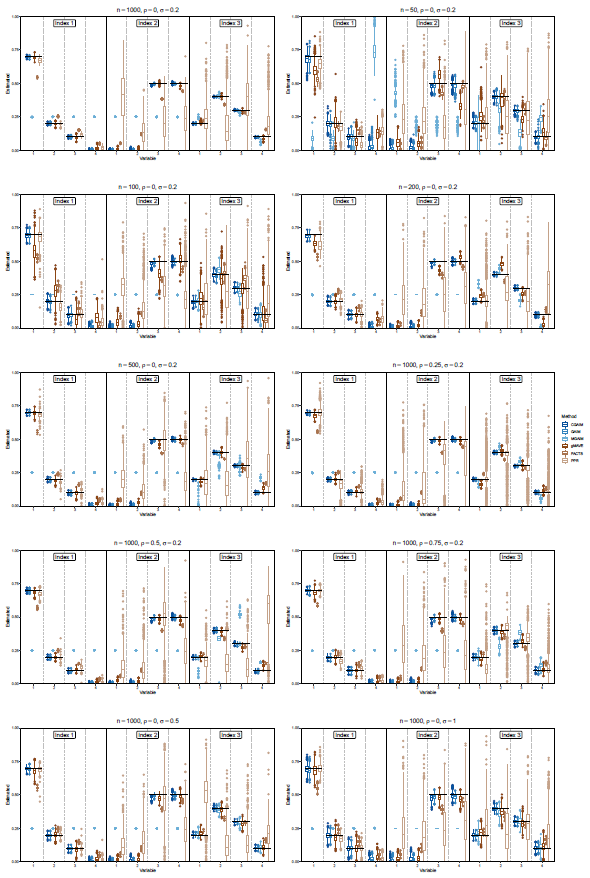


Supplementary Figure 3: Distribution of estimated $\hat{\alpha}_{j}$ for the 10 scenarios in experiment 1. Horizontal segments indicate the true $\boldsymbol{\alpha}_{j}$ used in data generation.

Supplementary Table 4: Average execution time and interquartile range across simulations for each model. Executed on a 3.20GHz CPU with 64GB RAM.

| **Model** | **Time (Interquartile range) in seconds** |
| --- | --- |
| CGAIM | 0.60 (0.44 - 0.75) |
| GAIM | 1.31 (0.51 - 1.74) |
| MGAIM | 0.28 (0.21 - 0.36) |
| gMAVE | 15.11 (1.91 - 22.92) |
| FACTS | 111.78 (4.36 - 167.08) |
| PPR | 0.01 (0.00 - 0.02) |


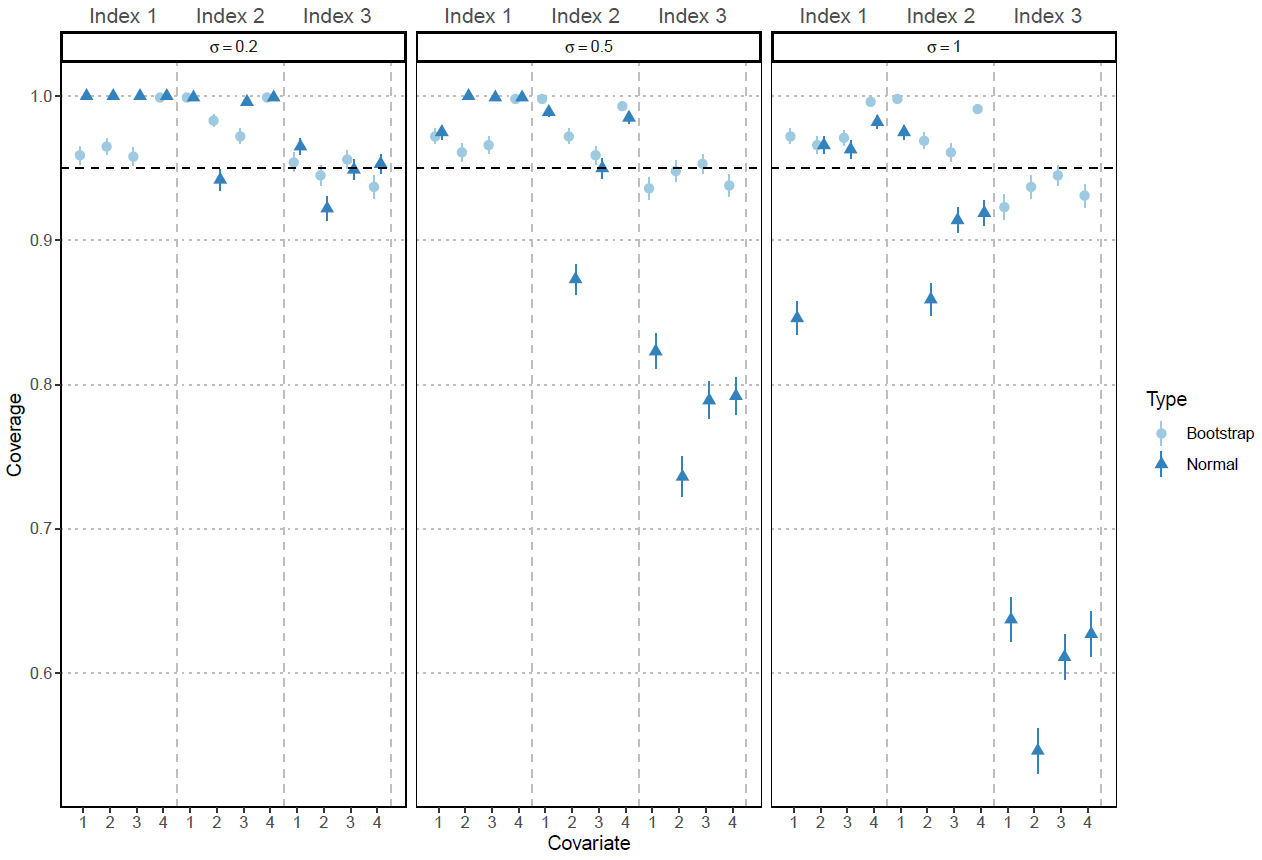


Supplementary Figure 4: Coverage disaggregated by covariate in experiment 3. Vertical segments indicate the +/- standard error range.

# Application to air pollution index

We present here an addition application concerned with the construction of an air quality index (AQI) in the city of Montreal, using data of the Canadian National Air Pollution Surveillance Network (NAPS). We consider same day nitrogen dioxide (NO_2_), ozone (O_3_) and fine particulate matter (PM_2.5_), the three most adverse pollutants (World Health Organization 2013). To obtain an index that predicts adverse health outcomes, we consider cardiovascular mortality as the outcome $Y$ in the CGAIM. The applied CGAIM is then

|  | $Y=\beta_{0}+\beta_{1}g(\alpha_{1}PM_{2.5}+\alpha_{2}O_{3}+\alpha_{3}NO_{2})+\gamma_{1}f_{1}\left. \left( DOS \right. \right)+\gamma_{2}f_{2}\left. \left( year \right. \right)+\epsilon$ | (9) |
| --- | --- | --- |

Note that since a single index is created here, we drop the coefficient $\beta_{1}$ in (9). Since a priori all the pollutants have a positive relationship with cardiovascular mortality, we add the constraint that all $\alpha_{j}\geq0$, sums to one and that $g$ is monotone increasing. We also apply an (unconstrained) GAIM as comparison. The AQI constructed seeks to find the right weight to give to each pollutant to best predict adverse effects.

Figure 5 shows the estimated weights $\boldsymbol{\alpha}$ of the AQI in the top panel and its relationship with cardiovascular mortality in the bottom panel. The largest weight is attributed to NO_2_, followed by O_3_ and PM_2.5_ that has a null weight, although all three weights display very large confidence intervals. We find here that weights allowed to NO_2_ and O_3_ are respectively 66 and 34 % which corresponds to weights usually attributed to compute their combined oxidative potential (Weichenthal *and others* 2016) meaning that there is theoretical support for these proportions. Weights attributed by the GAIM are slightly lower for NO_2_ and O_3_, although keeping a similar relative proportion between them, with a negative weight for PM_2.5_. Although this seems less realistic given the documented adverse effects of fine particulate matter (Liu *and others* 2019), pollutants are subject to many chemical reactions in the atmosphere, resulting in complex interactions potentially modifying their association with health. The estimated association is non-null for high levels of pollution, which means that the index is potentially useful for extreme events and facilitates the construction of alert thresholds.


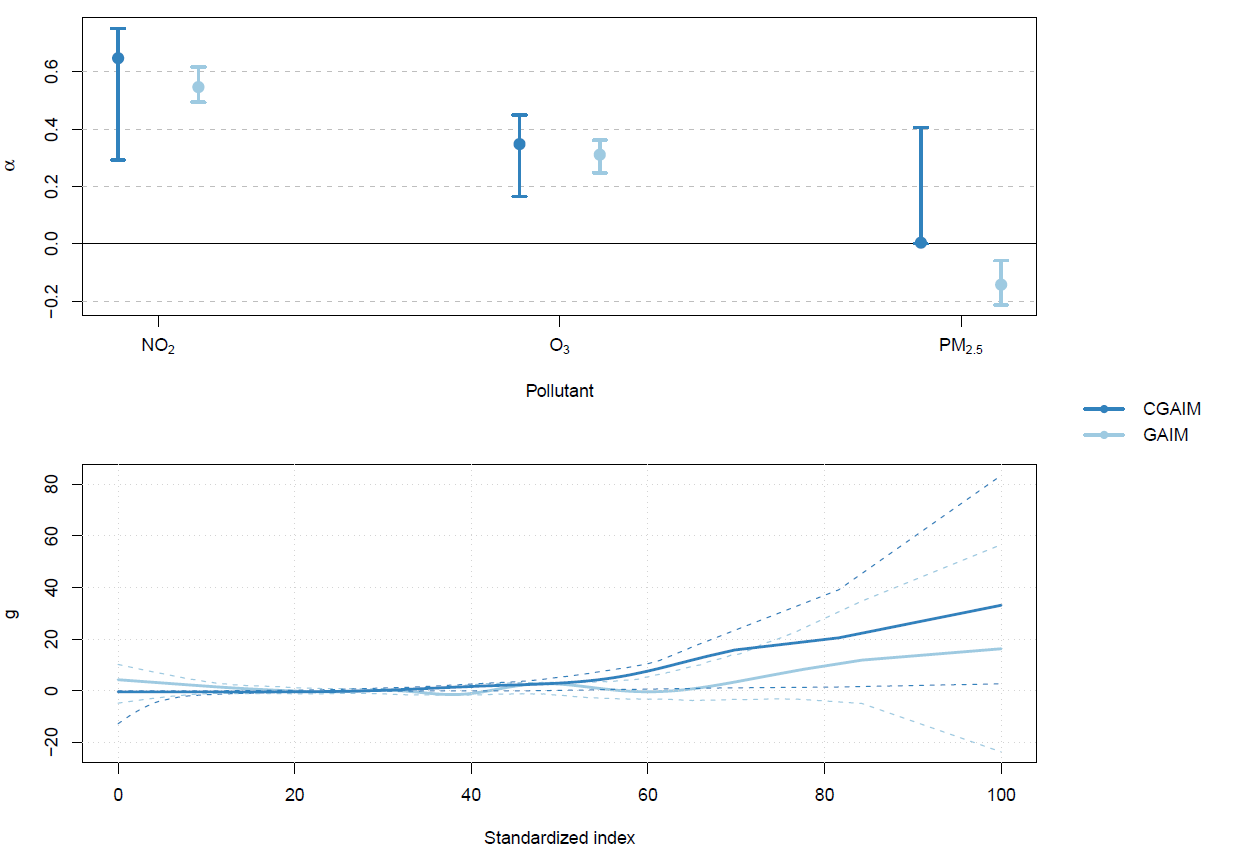


Supplementary Figure 5: Estimated AQI and relationship with the response. Segments in top panel and dotted lines in bottom panel indicate the 95% block bootstrap confidence intervals.

# References

Kong, E., Tong, H. and Xia, Y. (2010). Statistical modelling of nonlinear long-term cumulative effects. *Statistica Sinica* **20**, 1097–1123.

Li, L., Li, B. and Zhu, L.-X. (2010). Groupwise Dimension Reduction. *Journal of the American Statistical Association* **105**, 1188–1201.

Liu, C., Chen, R., Sera, F., Vicedo-Cabrera, A. M., Guo, Y., Tong, S., Coelho, M. S. Z. S., Saldiva, P. H. N., Lavigne, E., Matus, P., et al. (2019). Ambient Particulate Air Pollution and Daily Mortality in 652 Cities. *New England Journal of Medicine* **381**, 705–715.

Robinson, O., Tamayo, I., de, C. M., Valentin, A., Giorgis, -Allemand Lise, Hjertager, K. N., Marit, A. G., Ambros, A., Ballester, F., Bird, P., et al. (2018). The Urban Exposome during Pregnancy and Its Socioeconomic Determinants. *Environmental Health Perspectives* **126**, 077005.

Weichenthal, S., Lavigne, E., Evans, G., Pollitt, K. and Burnett, R. T. (2016). Ambient PM2.5 and risk of emergency room visits for myocardial infarction: impact of regional PM2.5 oxidative potential: a case-crossover study. *Environmental Health* **15**, 46.

World Health Organization. (2013). Review of evidence on health aspects of air pollution – REVIHAAP Project. Geneva, Switzerland: World Health Organization.
